# Intrageneric cross-reactivity of monospecific rabbit antisera against venoms of mamba (Elapidae: *Dendroaspis* spp.) snakes

**DOI:** 10.1016/j.toxcx.2023.100183

**Published:** 2024-01-04

**Authors:** Aarón Gómez, Andrés Sánchez, Gina Durán, Mauren Villalta, Álvaro Segura, Mariángela Vargas, María Herrera, Melvin Sánchez, José María Gutiérrez, Guillermo León

**Affiliations:** Instituto Clodomiro Picado, Facultad de Microbiología, Universidad de Costa Rica, San José, Costa Rica

**Keywords:** Cross-reactivity, Immunization, *Dendroaspis* spp, Snake antivenom, Snake venom, Sub-saharan africa

## Abstract

Snakebite envenomation is a neglected tropical disease posing a high toll of mortality and morbidity in sub-Saharan Africa. Polyspecific antivenoms of broad effectiveness and specially designed for this region require a detailed understanding of the immunological features of the mamba snake (*Dendroaspis* spp.) venoms for the selection of the most appropriate antigen combination to produce antivenoms of wide neutralizing scope. Monospecific antisera were generated in rabbits against the venoms of the four species of mambas. The toxic effects of the immunization scheme in the animals were evaluated, antibody titers were estimated using immunochemical assays, and neutralization of lethal activity was assessed. By the end of the immunization schedule, rabbits showed normal values of the majority of hematological parameters tested. No muscle tissue damage was noticed, and no alterations in most serum chemical parameters were observed. Immunological analyses revealed a variable extent of cross-reactivity of the monospecific antisera against the heterologous venoms. The venoms of *D. jamesoni* and *D. viridis* generated the antisera with broader cross-reactivity by immunochemical parameters. The venoms of *D. polylepis* and *D. viridis* generated the antisera with better cross-neutralization of lethality, although the neutralizing ability of all antisera was lower than 0.16 mg venom/mL antiserum against either homologous or heterologous venoms. These experimental results must be scaled to large animal models used in antivenom manufacture at industrial level to assess whether these predictions are reproducible.

## Highlights

• The monospecific rabbit sera against individual mamba venoms were raised.
• The cross-reactivity of monospecific sera was assessed by immunochemical tests.
• The antigenic relatedness among mamba snake venoms was assessed.
• The anti-*D. jamesoni* and anti-*D. viridis* showed the highest cross-reactivity.

## Graphical abstract

**Figure:**
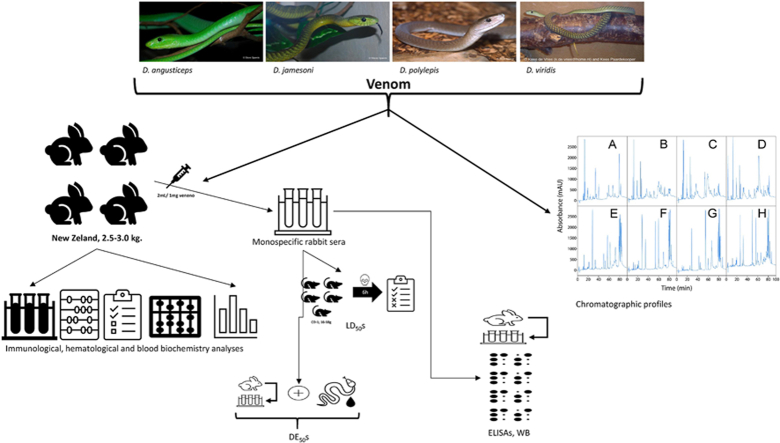

## 1 Introduction

The Elapidae snake family is widely distributed, with many species classified in category 1, i.e., species of highest medical importance, by the World Health Organization (WHO) (WHO, 2016). There are several elapid genera in Africa, including *Dendroaspis*, commonly known as mambas (Kelly et al., 2011; Pyron et al., 2011). *Dendroaspis* spp. Are endemic to sub-Saharan Africa and comprises four species: *D*. *angusticeps* (eastern green mamba), *D*. *jamesoni* (Jameson's mamba), *D*. *viridis* (western green mamba), all of them arboreal, and *D*. *polylepis* (black mamba), with terrestrial habits (Ainsworth et al., 2018; Chippaux and Jackson, 2019). These snakes are ambush predators with sedentary behaviors (Ainsworth et al., 2018; Chippaux and Jackson, 2019).

The proteomic composition of the venoms from mambas has been studied (Laustsen et al., 2015; Lauridsen et al., 2016; Petras et al., 2016; Ainsworth et al., 2018). They are mainly composed of non-enzymatic post-synaptically acting three-finger toxins (3FTx), and the pre-synaptically acting Kunitz-type serine proteinase inhibitor-like (KUN) toxins (i.e., dendrotoxins) (Ainsworth et al., 2018). Within the 3FTx family, there are toxins with diverse biological functions, such as antagonists of muscular nicotinic cholinergic receptors (short or long-chain post-synaptic α-neurotoxins) and muscarinic receptors (i.e., cardiotoxins known as muscarinic toxins), inhibitors of acetylcholinesterases which cause fasciculations (i.e., fasciculins), L-type calcium channels blockers that inhibit smooth muscle contraction and cardiac function (i.e., calciseptine), and inhibitors of acid-sensing ion channels (i.e., mambalgins) (Laustsen et al., 2015; Lauridsen et al., 2016; Petras et al., 2016; Ainsworth et al., 2018).

The dendrotoxins exert neurotoxic activity via inhibition of voltage-dependent K^+^ channels, causing a stimulatory effect resulting in the release of acetylcholine at the nerve terminals in neuro-muscular junctions (Harvey, 2001; Harvey and Robertson, 2004). Additionally, mamba venom contains other KUN toxins such as calcicludine (Schweitz et al., 1999), targeting voltage-dependent Ca^2+^ channels which are essential for controlling cardiac and smooth muscle contractions (Ainsworth et al., 2018). Less abundant toxin families were also detected in the venoms of mambas, such as snake venom metalloproteinases (SVMPs), natriuretic peptides (NP) (Park et al., 2012), mamba intestinal toxins (MIT) (Schweitz et al., 1999), and phospholipase A_2_ (PLA_2_) (Ibrahim and Masr, 1975).

Mamba envenomations in humans are characterized clinically by paresthesia, nausea, vomiting, abdominal pain, diarrhea, sweating, and hypersalivation (Závada et al., 2011). Additionally, neurotoxic manifestations include ptosis, diplopia, dysphagia, fasciculation, respiratory paralysis, and cardiovascular collapse, which could lead to death (Warrell, 1995; WHO, 2010; Závada et al., 2011). Unfortunately, fatality rates are high without mechanical ventilation and the rapid administration of antivenom (O'Shea, 2011).

Although antivenom administration is the only effective treatment against snakebite envenomation (WHO, 2010), there is limited preclinical information on the efficacy of antivenoms to bind relevant toxins and to neutralize the toxicity of *Dendroaspis* sp., venoms (Laustsen et al., 2015; Ainsworth et al., 2018; Ochola et al., 2019). Moreover, some findings have underscored the lack of preclinical efficacy of some polyspecific antivenoms against mamba venoms (Harrison et al., 2017).

The venom selection to fabricate polyspecific antivenoms should be based on the relationship between conservation and variation of the antigenic and immunogenic features of venoms from the snakes that pose a threat to public health and are medically important in the region where the antivenoms are intended to be used. Thus, the selection of appropriate venoms for immunization purposes should be done based on detailed knowledge of the medical relevance of the snake species and their venom immunological relatedness (WHO, 2010; León et al., 2011; Lauridsen et al., 2016; WHO, 2016; Ochola et al., 2019; Rathore et al., 2023). This represents a challenging task due to venom variation and species relationships within this genus (Ainsworth et al., 2018).

In this work, we determined the antigenic relatedness among venoms of the four species of mambas based on the intrageneric cross-reactivity of monospecific rabbit sera raised against individual mamba venoms. This information may be useful for the rational, knowledge-based design of appropriate venom mixtures for the generation of pan-African antivenoms.

## 2 Materials and methods

### Ethical statement

This work presents an experimental study conducted following the standard procedures of scientific ethics, including those relating to the use and care of animals. All procedures done in this study meet the International Guiding Principles for Biomedical Research Involving Animals (Bankowski and Howard-Jones, 1986). All procedures involving animals were approved by the Institutional Committee for the Care and Use of Laboratory Animals of Universidad de Costa Rica (approval code CICUA 202–2020). Rabbits and mice of both sexes were obtained from the Bioterium of Clodomiro Picado Institute. Rabbits were handled in Scanbur type EC3 cages (L 823 * W 660 * H 110 mm), one rabbit per cage, while mice were managed in Tecniplast Eurostandard Type II 1264C cages (L 25 * W 40 * H 14 cm), five mice per cage. In both cases, animals were kept at 18–24 °C, 60–65% relative humidity, and a 12:12 h light-dark cycle.

### 2.1 Snake venoms

Venoms of adult specimens of *Dendroaspis angusticeps* (Tanzania, Mozambique; batch #305.000), *D*. *jamesoni* (Cameroon; batch #923.011), *D*. *polylepis* (unknown origin; batch #416.031) and *D*. *viridis* (Ghana, Togo; batch #516.001) were purchased from Latoxan (Portes-dès Valence, France). Venoms were provided as freeze-dried preparations and were stored at −40 °C. Venom solutions were prepared immediately before use by reconstitution with the corresponding solvents, as described below.

### 2.2 Reverse-phase HPLC profiling

Five milligrams of each venom were dissolved in 200 μL of 0.1% trifluoroacetic acid (TFA) and 5% acetonitrile buffer (buffer A), the insoluble material was discarded after centrifugation, and the protein content in the supernatant was fractionated by reverse-phase HPLC (RP-HPLC, HPLC system: Agilent 1100 series; Agilent Technologies), equipped with a C18 column (250 × 4.6 mm, 5 μm particle size; Agilent Technologies). The flow rate was set to 1 mL/min and the protein separation was performed with the following buffer gradient: 0% buffer B (buffer B: 95% acetonitrile, 0.1 % TFA) for 5 min, followed by 0–15% B over 10 min, 15–45% B over 60 min, 45–70% B over 10 min and 70% B for 9 min (Lomonte and Calvete, 2017). Protein peaks were detected at 215 nm. The composition of HPLC fractions was inferred by comparing the chromatograms with those previously published.

### 2.3 Determination of lethal activity

Groups of five mice (16–18 g; CD-1 strain, both sexes) were injected subcutaneously (SC) with the analgesic Tramadol (50 mg/kg) to reduce pain during the test (Herrera et al., 2018). Fifteen minutes afterward, mice received an intraperitoneal (IP) route of 0.5 mL of 0.12 M NaCl, 0.04 M phosphate, pH 7.2, solution (PBS) containing different amounts of venom. The number of deaths occurring during the following 6 h was recorded (Durán et al., 2021) and used to estimate the median lethal dose (LD_50_, i.e., the amount of venom that results in the death of 50% of the injected mice) by Probits (Finney, 1971). The observation time of 6 h, instead of 48 h, is justified for ethical considerations, and on the basis of previous observations (Durán et al., 2021). Surviving mice were euthanized by CO_2_ inhalation. Results were reported as LD_50_ and the corresponding 95% confidence interval (95% CI).

### 2.4 Immunization of rabbits

Groups of four rabbits (New Zealand, 2.5–3.0 kg body weight, both sexes) were immunized with the venoms of individual mamba species (i.e., *D*. *angusticeps*, *D*. *jamesoni*, *D*. *polylepis*, or *D*. *viridis*). Immunization was performed by five SC injections applied at two-week intervals. The total volume of injections was 2 mL, and all of them contained 1 mg of venom. The venom was dissolved in 1 mL of sterile saline solution, added to 1 mL of Montanide® adjuvant and emulsified before application. All the injections were prepared using the emulsified Montanide® solution. Rabbit health monitoring was performed by a veterinarian during the immunization. At the end of the immunization, samples of blood (6 mL) were collected from the ear marginal vein, where 3 mL were added to EDTA as anticoagulant and used for hematological test and the other 3 mL were left to clot to obtain the serum used for blood chemistry and immunological analyses. Rabbits were then euthanized by an overdose of anesthetic (i.e., a dose of 100 mg/kg of sodium pentobarbital, administered by the IP route).

### 2.5 Hematological and serum chemistry analysis

Hematological and serum chemistry analyses were conducted on each individual rabbit sample. Hematological analyses (see Supplementary Table S1 for the list of parameters analyzed) were conducted in a Veterinary Hematology Analyzer (Exigo Eos Hematology System; Boule Diagnostics AB, Stockholm, Sweden). The following analytes were quantified in a clinical chemistry analyzer (Spin200 E Automatic biochemistry analyzer; Spinreact, Barcelona, España): creatine kinase (CK), aspartate aminotransferase (AST), alanine aminotransferase (ALT), and alkaline phosphatase (ALP), determined by the corresponding International Federation of Clinical Chemistry and Laboratory Medicine (IFCC) methods. Urea was quantified by a modification of the Talke and Schubert (1965) method; creatinine by a kinetic modification of the Jaffe colorimetric method (Mazzachi et al., 2000); total protein by the Biuret method (Gornall et al., 1949) and albumin by the bromocresol green colorimetric method (Rodkey, 1965).

### 2.6 Immune reactivity of rabbit sera by enzyme-linked immunosorbent assay (ELISA)

Polystyrene plates were coated overnight at room temperature with 100 μL of PBS containing 3 μg of venom. After washing the plates five times with distilled water, 100 μL of several dilutions of each rabbit serum sample (dilution factor 3; from 1:1500 to 1:40,500), in PBS-2% bovine serum albumin (BSA), were added. Plates were incubated for 1 h at room temperature and washed five times. Afterward, 100 μL of goat anti-rabbit IgG conjugated with peroxidase (Sigma-Aldrich A0545), diluted 1:5000 with PBS-2% BSA, were added to each well. Microplates were incubated for 1 h at room temperature. After a final washing step, color was developed by the addition of H_2_O_2_ and *o*-phenylenediamine. Color development was stopped by the addition of 1.0 M HCl. Absorbances at 492 nm were recorded. The relative concentration of anti-venom antibodies in the samples was calculated by interpolation of their absorbances in a calibration curve. Relative concentration was expressed as a percentage, 100% corresponding to the titer of the serum raised against the homologous venom of each species. Results were expressed as mean ± SD of all rabbits in each group.

### 2.7 Electrophoretic analysis and western blot

Venoms (30 μg) were separated by SDS-PAGE and run under non-reducing conditions using an acrylamide concentration of 12 % (Laemmli, 1970), using the Millipore mPAGE Color (MPSTD4) molecular marker (3 μL). The gels were stained with Coomassie Brilliant Blue R-250, decolored with water and used to display the electrophoretic venom profiles, or transferred to a nitrocellulose membrane at 30 mAmp overnight. The gels used for Western blot were later stained using Coomassie Brilliant Blue R-250 to confirm the protein transfer to the membranes. The membranes were blocked with PBS-0.1% casein for 30 min. Next, membranes were incubated for 1 h with a pool of serum samples of all rabbits of each monospecific antiserum, diluted 1/1000 with PBS-0.1% casein. After washing the membranes three times with PBS-0.1% casein, they were incubated for 1 h with goat anti-rabbit IgG (Sigma A4914) conjugated with alkaline phosphatase, diluted 1:2000 with PBS-0.1% casein. Finally, after the last washing step, a 1-chloro-4-naftol color development substrate was added in the presence of methanol and peroxide hydrogen, and the reaction was stopped with distilled water.

### 2.8 Neutralization of the lethal activity

The ability of serum pooled samples from all rabbits in each group to neutralize the lethal activity of the venoms was assessed by mixing a constant challenge dose of each venom with different dilutions of the pool of each antiserum. Mixtures were incubated at 37 °C for 30 min before determining the residual activity of venom by using the experimental system described above. The challenge dose utilized was 2 LD_50_s. In all cases, venom-only controls were included, in which venom was incubated with PBS instead of antiserum. The use of 2 LD_50_s as a challenge dose in the neutralization of lethality studies, instead of the usual 4–5 LD_50_s, is justified to increase the sensitivity of the assay, favoring the detection of cross-reactivity of antisera against venoms. The neutralization was expressed as the median effective dose (ED_50_), defined as the ratio of mg venom/mL antiserum at which 50% of injected mice survived (Segura et al., 2010). Results were reported as ED_50_ and the corresponding 95% confidence interval (95% CI).

### 2.9 Statistical analysis

The hematological parameters and plasma chemistry analytes were assessed by one-way ANOVA, followed by Dunnett's post-hoc test. For ELISA's assays, the significance of the differences between mean values of groups was assessed by one-way ANOVA, followed by a Ryan-Einot-Gabriel-Welsch Range (R-E-G-W Q) post-hoc test. In the case of lethality neutralization, groups having non-overlapping values of 95% CI were considered significantly different. Finally, linearity and homogeneity of variances were assessed, and a *p*-value <0.05 was considered significant.

## 3 Results

**Fig. 1:**
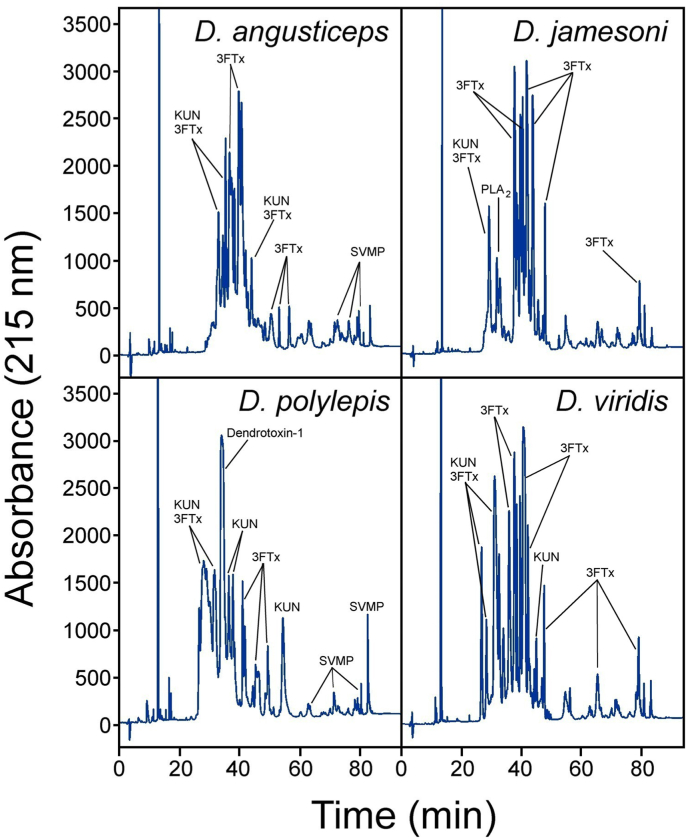
RP-HPLC chromatograms of mamba venoms. The composition of HPLC fractions was inferred by comparing the chromatograms with previously published data for *D. angusticeps* (Lauridsen et al., 2016; Petras et al., 2016; Ainsworth et al., 2018), *D. jamesoni* (Ainsworth et al., 2018), *D. polylepis* (Laustsen et al., 2015; Petras et al., 2016; Ainsworth et al., 2018) and *D. viridis* (Ainsworth et al., 2018). KUN: Kunitz-type proteinase inhibitor, 3FTx: three-finger toxin; PLA2: phospholipase A2; SVMP: snake venom metalloproteinase.

The chromatographic venom profiles of the four mamba species were obtained (Fig. 1), and intrageneric variations in the relative abundance of the fractions were noticed, as expected on the basis of previously published data for *D*. *angusticeps* (Lauridsen et al., 2016; Petras et al., 2016; Ainsworth et al., 2018), *D*. *jamesoni* (Ainsworth et al., 2018), *D*. *polylepis* (Laustsen et al., 2015; Petras et al., 2016; Ainsworth et al., 2018) and *D*. *viridis* (Ainsworth et al., 2018). Overall, the composition of the venoms is consistent with the toxicological profile, showing a high content of three-finger toxins (3FTxs) and Kunitz-type inhibitors (KUN), which are the major venom components that cause neurotoxicity. On the other hand, some family proteins, such as PLA_2_, are present in *D*. *jamesoni* but absent or in very low quantity in *D*. *angusticeps*, *D*. *polylepis* and *D*. *viridis* (Fig. 1). Additionally, the venoms showed differences in their LD_50_s. Venoms having the highest toxicity were *D*. *polylepis* and *D*. *viridis*; yet all venoms were highly toxic (Table 1).

**Table 1:** Venom toxicity of *Dendroaspis* sp.

Venom	Lethality μg/g (95% CI)	Lethality μg/16–18 g mouse (95% CI)
*D. angusticeps*	1.27 (0.93–1.79)	21.7 (15.8–30.5)
*D. jamesoni*	1.08 (0.83–1.55)	18.5 (14.2–26.5)
*D. polylepis*	0.38 (0.28–0.50)	6.6 (4.9–8.6)
*D. viridis*	0.43 (0.32–.074)	7.3 (5.5–12.6)

Lethality is expressed as LD_50_ (95% CI) by the i.p. route; i.e., the Median Lethal Dose, defined as the amount of venom that results in the death of 50% of the injected mice (16 g–18 g).

Groups of four rabbits were immunized with each of the four mamba venoms. The immunization scheme was applied using Montanide® adjuvant emulsified with sterile saline solution (Arguedas et al., 2022), allowing the emulsification of venom in Montanide® being slowly released and reducing the potential risk of tissue damage and overall toxicity, enhancing the antibody response of the animal. By the end of the immunization program, the rabbits showed normal hematological values except for the mean corpuscular volume (MCV; F_(4; 14)_ = 414.055, *p* < 0.001), the red blood cell distribution width (RDW; F_(4; 14)_ = 3.435, *p* = 0.037), the monocytes (F_(4; 14)_ = 3.268, *p* = 0.043) and the platelets (F_(4; 14)_ = 3.187, *p* = 0.047), when compared to the control group. These alterations may be secondary to the inflammatory response induced by the venom (Supplementary Table S1).

Additionally, no muscle tissue or hepatic damage was evidenced during the immunization since the plasma levels of CK, AST, ALT, and ALP in immunized animals were similar to those of the control group of non-immunized animals (Supplementary Table S2). Moreover, no signs of kidney damage were observed, with the exception of an increment in the urea serum concentration (F_(4; 14)_ = 3.130, *p* = 0.049) of rabbits immunized with the venom of *D*. *angusticeps* (Dunnett *t*-test *p* = 0.019) when compared to the control group (Table S2).

**Fig. 2:**
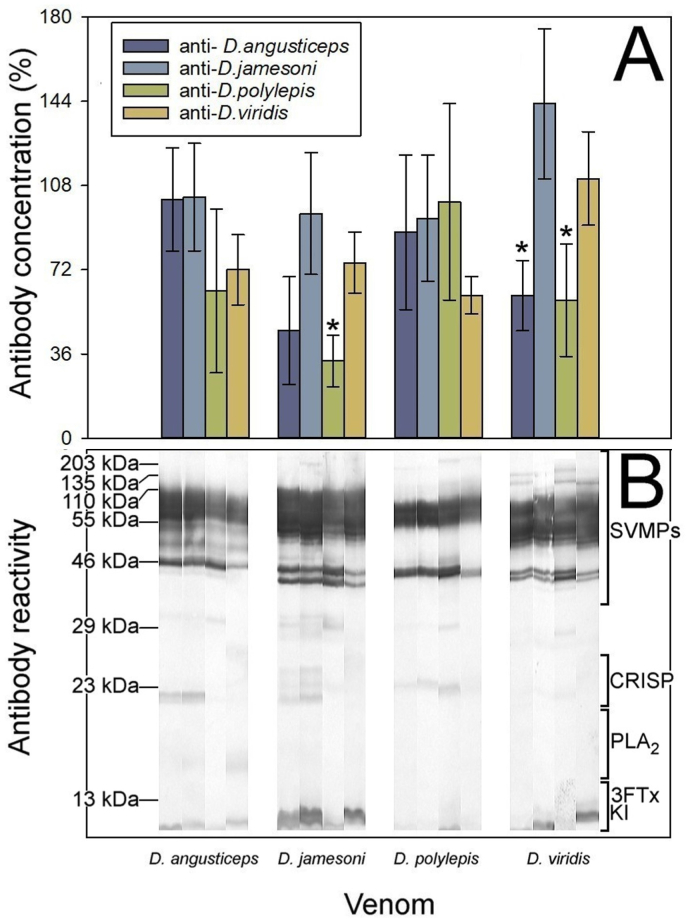
Cross-reactivity between the mamba venoms was determined by A) ELISA and B) Western blot. ELISA results are expressed as percentage, considering as 100% the titer of serum raised against the homologous venom of each species and correspond to the mean ± SD of all rabbits in each group. *Show differences (*p* < 0.05) in antibody concentration of the heterologous antisera compared to the homologous antiserum.

The experimental protocol used by Gómez and colleagues (Gómez et al., 2022, 2023) was followed to assess the cross-reactivity and cross-neutralization of the antisera, using a combination of antibody titers quantified by ELISA, Western blot, and neutralization of the lethal activity to assess the intrageneric cross-reactivity of monospecific rabbit sera against homologous and heterologous venoms. Cross-reactivity was evidenced in all the anti-*Dendroaspis* antisera against the four mamba venoms studied, showing an antigenic similarity between venoms, yet quantitative differences were noticed (Fig. 2). No differences in the cross-recognition by ELISA were observed between the monospecific antisera and the homologous and heterologous venoms for *D*. *angusticeps* (F_(3; 10)_ = 2.334, *p* = 0.136) and *D*. *polylepis* (F_(3; 10)_ = 1.007, *p* = 0.430), whereas differences in the cross-recognition for the antisera against the homologous and heterologous *D*. *jamesoni* (F_(3; 10)_ = 7.993, *p* = 0.005) and *D*. *viridis* (F_(3; 10)_ = 10.671, *p* = 0.002) venoms were observed. The venom of *D*. *jamesoni* was poorly recognized by the anti-*D*. *polylepis* when compared to the homologous antiserum (R-E-G-W Q test *p* = 0.060), while the venom of *D*. *viridis* was also poorly recognized by the anti-*D*. *angusticeps* and the anti-*D*. *polylepis* antisera (R-E-G-W Q test *p* = 0.996) when compared to the homologous antiserum (Fig. 2A). As a general trend, the anti-*D. jamesoni* and anti-*D. viridis* antisera provided the highest level of cross-reactivity by ELISA.

In addition, cross-reactivity was also assessed by Western blot, and the identification of the immunoreactive protein bands was based on the molecular masses. In general, the antisera showed a high immunoreactivity against proteins of high molecular masses (>40 kDa), which are known to have low toxicity in these venoms. In contrast, recognition of bands where 3FTxs and dendrotoxins migrate was generally weaker and, in some cases, recognition was almost absent (Fig. 2B). For example, antiserum against *D. polylepis* showed a poor recognition of low molecular mass bands of the heterologous venoms, while these proteins in the venom of *D. polylepis* were poorly recognized by heterologous antisera (Fig. 2B). The strongest immunoreactivity in the low molecular mass region of the venoms was observed with the *D. jamesoni* and *D. viridis* antisera (Fig. 2B).

**Fig. 3:**
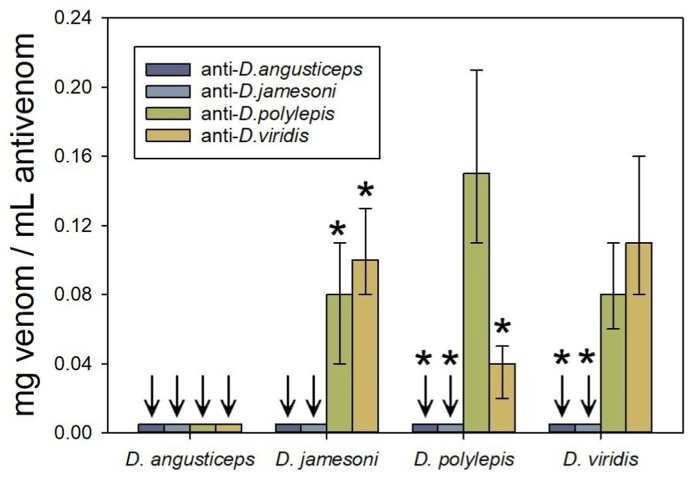
Neutralization of lethality of venoms by monospecific antisera (see materials and methods for details). Neutralization is expressed as the median effective dose (ED_50_), i.e., the ratio mg venom/mL antivenom at which 50% of the injected mice are protected. Bars correspond to the 95% confidence intervals. * Significantly different as compared to the homologous antisera. ↓ values under the detection limit of the lethality neutralization assay.

A poor lethality neutralization was found with all the homologous and heterologous antisera evaluated, since all of them had ED_50_ values lower than 0.16 mg venom/mL antiserum (Fig. 3). The antisera against *D. angusticeps* and *D. jamesoni* failed to neutralize the four venoms assessed. Antisera against *D. polylepis* and *D. viridis* were effective in the neutralization of the venoms of *D. jamesoni*, *D. polylepis* and *D. viridis*, but failed in the neutralization of the *D*. *angusticeps* venom (Fig. 3).

## 4 Discussion

In general, the venoms analyzed by their chromatographic profiles showed similar composition to those previously published (Laustsen et al., 2015; Lauridsen et al., 2016; Petras et al., 2016; Ainsworth et al., 2018), with a predominance of low molecular mass neurotoxic components. Thus, although clinical effects of human envenomations by these species may differ, neurotoxic manifestations predominate (WHO, 2010). The antivenoms used as treatment must be able to effectively neutralize the neurotoxic clinical effects induced by all the species.

The venom of the mambas analyzed showed similar LD_50_s (Table 1), and similar values have been published (Ainsworth et al., 2018), even though the administration route used was different, underlining the high toxicity of these venoms. This high toxicity is attributed to the presence of dendrotoxins, 3FTxs protein family including α – neurotoxins, muscarinic toxins and fasciculins (Laustsen et al., 2015; Lauridsen et al., 2016; Petras et al., 2016; Ainsworth et al., 2018), conferring the venoms with high neurotoxicity.

On the other hand, it was of interest to assess the potential toxicity induced by mamba venoms in this rabbit model used for immunization. On the basis of hematological and plasma biochemistry analyses, it is suggested that no significant tissue damage was induced during the immunization. This agrees with the known pathophysiological profile of envenomings by mamba venoms, which are mainly characterized by prominent neurotoxicity (Warrell, 1995).

It might be beneficial to consider an improvement of the biochemical plasma analyses when the immunization effects are being assessed for neurotoxic activities by including the determination of acetylcholinesterase (butyrylcholinesterase) plasma activity assay, which could be helpful to assess the effect of fasciculins (Petras et al., 2016), which are cholinesterase inhibitors, in immunized animals.

When the cross-recognition and neutralization between *Dendroaspis* spp. Venoms were assessed, the data from the antibody recognition by Western blot and the neutralization of lethality highlight the difficulty in generating high antibody titers against relevant neurotoxins of mamba venoms. Nevertheless, the cross-reactivity between monospecific anti-mamba antisera reveals antigenic similarities between the components of all mamba species. It was of interest that antisera raised against the venoms of *D. jamesoni* and *D. viridis* showed the highest cross-reactivity by ELISA and also the highest recognition of low molecular mass bands in Western blot, where neurotoxins migrate. However, although *D. viridis* antiserum neutralized the lethal activity of venoms from three species, *D. jamesoni* antivenom was ineffective. Further insights into this phenomenon can be accomplished through high-throughput technologies (i.e., antivenomics and toxicovenomics) and novel in-vitro assays (i.e., cell bioassays) (Laustsen et al., 2015; Lauridsen et al., 2016; Petras et al., 2016; Ainsworth et al., 2018; Patel et al., 2023).

Preclinical studies of commercially available antivenoms, using lethality neutralization assays and antivenomics, have revealed marked differences between antivenoms in their ability to bind neurotoxins or neutralize the lethality of venoms (Laustsen et al., 2015; Harrison et al., 2017; Ainsworth et al., 2018; Ochola et al., 2019). In addition, there is a need to develop novel strategies for improving antivenom-neutralizing ability, such as using more effective adjuvants, neurotoxic fractions for immunization, or concentrating the anti-neurotoxin antibodies in the final product.

## 5 Conclusions

The main aim of this study was to identify the venoms of *Dendroaspis* species that would generate an antibody response with the widest cross-reactivity and cross-neutralization within the genus. If judged by ELISA results, the antisera against *D. jamesoni* and *D*. *viridis* showed the highest cross-reactivity. Moreover, these antisera recognized proteins by Western blot in the molecular mass range where 3FTxs and dendrotoxins migrate. On this basis, these venoms are candidates to be used for generating cross-reacting antivenoms in large animal models used in antivenom production.

Although the neutralization of lethality is the gold standard to judge the efficacy of antivenoms, the low neutralizing ability of the monospecific antisera against homologous and heterologous venoms makes it difficult to select the most appropriate venoms for immunization on the basis of neutralization of lethality data. In addition, the geographical variation of venoms should be considered, and more studies are required with venoms from various geographical locations in Africa to establish the best combination of mamba venoms to produce antivenoms.

Finally, as immunogenicity depends on the nature of the immune system of the animal model selected as immunoglobulin source, the conclusions of our experiments in rabbits cannot be directly extrapolated to other animal models used in antivenom fabrication, such as horses or sheep. Nonetheless, these results can be applied to formulate rational hypotheses that can be put to experimental evaluation by immunizing horses with mixtures of mamba venoms to produce broad neutralizing antivenoms for sub-Saharan Africa.

## CRediT authorship contribution statement

**Aarón Gómez:** Writing - review & editing, Writing - original draft, Resources, Methodology, Investigation, Formal analysis, Data curation, Conceptualization. **Andrés Sánchez:** Writing - review & editing, Methodology, Investigation. **Gina Durán:** Writing - review & editing, Methodology, Investigation. **Mauren Villalta:** Writing - review & editing, Methodology, Investigation. **Álvaro Segura:** Writing - review & editing, Methodology, Investigation. **Mariángela Vargas:** Writing - review & editing, Methodology, Investigation. **María Herrera:** Writing - review & editing, Methodology, Investigation. **Melvin Sánchez:** Writing - review & editing, Methodology, Investigation. **José María Gutiérrez:** Writing - review & editing, Resources, Project administration, Funding acquisition, Formal analysis, Conceptualization. **Guillermo León:** Writing - original draft, Resources, Project administration, Funding acquisition, Formal analysis, Conceptualization.

## Declaration of competing interest

The authors declare the following financial interests/personal relationships which may be considered as potential competing interests: Guillermo León reports financial support and article publishing charges were provided by 10.13039/100010269Wellcome Trust. José María Gutiérrez reports financial support was provided by 10.13039/100010269Wellcome Trust. Guillermo León reports financial support was provided by Research Foundation of the 10.13039/501100005298University of Costa Rica. If there are other authors, they declare that they have no known competing financial interests or personal relationships that could have appeared to influence the work reported in this paper.

## Data Availability

Data will be made available on request.
