# Supplementary material for: Intrageneric cross-reactivity of monospecific rabbit antisera against venoms of mamba (Elapidae: Dendroaspis spp.) snakes
Source: Toxicon X. 2024 Jan 4;21:100183. doi: 10.1016/j.toxcx.2023.100183 (PMC10808963; doi:10.1016/j.toxcx.2023.100183)
Supplement: Multimedia component 1 [file mmc1.docx]

S1 Table. Hematological parameters of rabbit groups immunized with *Dendroaspis* sp venoms. Results are provided as mean ± SD.

| Species | RBC (x10^6^/µl) | MCV (fL) | RDW (%) | RDW (a) | Hct (%) | Hgb (g/dL) | MCH (pg) | MCHC (%) | WBC (x10^3^/µl) | LYM (%) | MONO (%) | GRAN (%) | PLT (x10^3^/µl) | Mpv (x10^3^/µl) |
| --- | --- | --- | --- | --- | --- | --- | --- | --- | --- | --- | --- | --- | --- | --- |
| *D. angusticeps* | 5.7 ± 0.2 | 66.7 ± 1.8* | 15.1 ± 0.7* | 39.9 ± 2.1 | 38.4 ± 0.9 | 12.3 ± 0.4 | 21.3 ± 0.6 | 32.0 ± 0.4 | 5.6 ± 1.8 | 38.0 ± 3.7 | 9.8 ± 2.1* | 52.1 ± 3.5 | 229.0 ± 100.2* | 3.6 ± 0.3 |
| *D. jamesoni* | 6.2 ± 0.6 | 66.2± 2.7* | 15.0 ± 1.4* | 39.5 ± 1.6 | 41.0 ± 4.7 | 13.0 ± 1.3 | 21.1 ± 1.0 | 31.9 ± 0.5 | 5.7 ± 0.8 | 33.8 ± 5.9 | 9.9 ± 0.7 | 56.2 ± 6.0 | 261.5 ± 67.7 | 3.8 ± 0.3 |
| *D. polylepis* | 5.9 ± 0.4 | 65.7± 2.9* | 15.6 ± 0.8 | 40.1 ± 1.8 | 39.1 ± 2.9 | 12.5 ± 0.9 | 21.0 ± 1.2 | 31.9 ± 0.6 | 7.5 ± 1.7 | 36.6 ± 6.0 | 10.5 ± 0.5* | 52.8 ± 5.9 | 225.7 ± 87.7* | 3.9 ± 0.2 |
| *D. viridis* | 6.2 ± 0.3 | 66.0 ± 2.8* | 14.5 ± 0.4* | 38.4 ± 1.7 | 41.0 ± 0.4 | 13.2 ± 0.3 | 21.1 ± 0.4 | 32.0 ± 0.8 | 7.1 ± 1.8 | 36.6 ± 5.3 | 10.8 ± 0.6* | 52.6 ± 5.7 | 221.0 ± 151.4* | 4.1 ± 0.5 |
| Control | 5.9 ± 1.5 | 62.0 ± 5.2 | 18.1 ± 2.6 | 41.1 ± 7.3 | 36.5 ± 6.8 | 11.9 ± 2.4 | 20.2 ± 1.4 | 32.6 ± 0.5 | 6.1 ± 1.7 | 36.2 ± 7.2 | 9.3 ± 1.6 | 54.4 ± 5.9 | 425.7 ± 136.1 | 3.9 ± 0.2 |

*Significantly different (*p*< 0.05) when compared to the control group. **RBC**: Red Blood Cells; **MCV**: Mean Corpuscular Volume; **RDW**: Red Blood Cell Distribution Width; **Hct**: Hematocrit; **Hgb**: hemoglobin; **MCH**: Mean Corpuscular Hemoglobin; **MCHC**: Mean Corpuscular Hemoglobin Concentration; **WBC**: White Blood Cells; **LYM**: Lymphocytes; **MONO**: Monocytes; **GRAN**: Granulocytes; **PLT**: Platelets; **MPV**: Mean Platelet Volume.

S2 Table. Biochemical parameters of rabbit groups immunized with *Dendroaspis* sp venoms. Values are compared to the control group and range reference values are provided. Results are presented as mean ± SD.

| SPECIES | | CK  (U/L) | AST  (U/L) | ALT  (U/L) | ALP  (U/L) | UREA  (mmol/L) | Creatinine  (µmol/L) | TP  (g/L) | Albumin  (g/L) |
| --- | --- | --- | --- | --- | --- | --- | --- | --- | --- |
| *D. angusticeps* | | 1272 ± 185 | 23 ± 6 | 40 ± 7 | 108 ± 29 | 9 ± 2* | 92 ± 7 | 71 ± 4 | 43 ± 1 |
| *D. jamesoni* | | 1128 ± 476 | 34 ± 17 | 71 ± 37 | 113 ± 40 | 8 ± 1 | 90 ± 12 | 66 ± 2 | 42 ± 1 |
| *D. polylepis* | | 1241 ± 286 | 25 ± 2 | 47 ± 14 | 122 ± 46 | 7 ± 2 | 94 ± 13 | 64 ± 4 | 41 ± 3 |
| *D. viridis* | | 1664 ± 709 | 26 ± 7 | 37 ± 8 | 120 ± 41 | 8 ± 2 | 86 ± 21 | 67 ± 4 | 42 ± 1 |
| Control | | 1137 ± 654 | 24 ± 6 | 49 ± 9 | 80 ± 53 | 6 ± 1 | 82 ± 3 | 60 ± 8 | 38 ± 4 |
| Reference | UL | 1.63 | 14 | 14 | 4 | 5.35 | 44.2 | 54 | 25 |
|  | LL | 559.53 | 113 | 80 | 70 | 17.85 | 229.84 | 75 | 50 |

*Significantly different (*p*< 0.05) when compared to the control group. **ALT**: alanine aminotransferase, **AST**: aspartate aminotransferase, **ALP**: alkaline phosphatase, **CK**: creatine kinase, **TP**: total protein. UL: Upper limit, LL: lower limit.
